# Impact of Spirulina Chikki Supplementation on Nutritional Status of Children: An Intervention Study in Tumkur District of Karnataka, India

**DOI:** 10.3389/fped.2022.860789

**Published:** 2022-04-15

**Authors:** Gyan Chandra Kashyap, R. Sarala, Usha Manjunath

**Affiliations:** Institute of Health Management Research, Bangalore, India

**Keywords:** nutritional status, anthropometric measurements, MUAC, spirulina, Tumkur, India

## Abstract

**Objective:**

To assess the impact of Spirulina Chikki supplementation on the nutritional status of children (6 months−6 years).

**Design:**

A cross-sectional study design was adopted to assess the changes in nutritional status among the children (after 12 months of intervention period). The bassline and endline assessment were carried out from September 2020 to August 2021, respectively.

**Setting:**

Total 106 villages (108 Anganwadi Centers in nine circles) from Tumkur District were covered.

**Methods:**

Children aged 6 months−6 years were the study subjects. Anthropometric measurements viz., height, weight, and mid-arm circumference were collected from total 971 and 838 children during baseline and endline assessments correspondingly. The information on children's health and nutrition status was gathered from the mothers of sampled children. WHO's Anthro and AnthroPlus software were utilized to estimate the anthropometric measurements (Stunting, wasting, and underweight) of study participants.

**Results:**

The study found apparent disparities in the prevalence of stunting, wasting and underweight among the male and female children. There was a significant decline viz., 4% (28.6%-baseline to 24.5%-end line) in the prevalence of severe wasting. Also, severe stunting dropped by 6% at end line (30%-end-line to 24%-baseline). Improvement in nutritional status was evident among both female male children in all three indicators stunting, wasting, and underweight. Mid-upper arm circumference (MUAC) measurement shows substantial improvements from baseline to end line: SAM (5.3–0.6%), MAM (23–9%), and normal (72–91%). The study discloses significant improvements in the nutritional status among those children who consumed spirulina chikkis/granules for a longer duration viz., 9–10 months as compared to those who consumed for lesser duration.

**Conclusions:**

Findings reveal improvement in nutritional status among the beneficiaries who consumed spirulina chikki/granules as per the recommended quantity (amount) during the intervention period. Post intervention, spirulina chikki supplementation for nutritional intervention is implied to address large scale malnutrition among young children.

## Introduction

Health and nutrition are the critical for the growth and human development. Since birth, better nutrition is of utmost importance for a child to have a robust immune system. It is essential to lower the chances of acquiring communicable and non-communicable diseases (1). Any form of malnutrition has significant threats to human health. At present, globally, especially in low- and middle-income countries double burden of malnutrition viz., both undernutrition (wasting, stunting, and underweight) and overnutrition are rising immensely (2). It is estimated that by 2020 globally, among the under five children, stunted, wasted, and overweight or obese children would be 149, 45, and 38.9 million, respectively (3).

India is the home to a sizable number of malnourished children across the world. According to NFHS-5 India, more than one-third of the under-five children (35.5%) were reported stunted, 19.3% were wasted (7.7% were severely wasted), and a little less of one-third (32.1%) were underweight. Further, it is evident that, the burden of malnutrition is high among the rural population in Tumkur. The most recent estimate exhibits that prevalence of stunting is high in Tumkur district (40.3%) as compared to it's the state (35.4%). This is an alarming situation since the prevalence of stunting is also higher than the national average (35.5%). Substantial change was observed in wasting in Tumkur District (10.9%) and Karnataka State (19.5%). Also, there is considerable improvement in severe wasting in Tumkur district (3.1%), and in the State (8.4%) (4–6).

Since independence through various national programmes including ICDS program, a considerable improvement in nutrition and well-being of children have been evident in India (7). Despite these improvements, malnutrition remains a critical issue of concern where India confers one-third of the global burden for undernutrition. To improve the nutritional status of the children and mothers, the National Nutritional Strategy provides a comprehensive platform for stakeholders to converge together and drive the agenda of “Mission Malnutrition Free India-2022” (8). Also, nutritional issue is a focused concern of Sustainable Development Goals (SDGs) no 12. Notably, the UN has acted on nutrition, offering a robust, joint country-driven program for all nutrition stakeholders to increase visibility, coordination, efficiency, and effectiveness of policy processes and activities across sectors at the national level to achieve the existing global nutrition targets by 2025 and the nutrition-related targets in the Agenda for Sustainable Development by 2030 (9).

Thus, aligning with the national goal to address the malnutrition in the country, Spirulina Foundation has trained and empowered local SHG women groups to cultivate spirulina and make Spirulina Fortified Chikkis (SFCs)/Granules in Kolar district of Karnataka. Spirulina Foundation has obtained approval from Central Food Technological Research Institute (CFTRI) Mysore for the processing of Spirulina chikkis/granules. Spirulina has superior macronutrient and micronutrient contents, and rich in amino acids, unsaturated fatty acids, B12, Provitamin A (β carotene), Vitamin E and Minerals, especially iron. It is also rich in gamma linolenic acid (GLA), an omega 3 fatty acid (10). Spirulina consists of 55–70% protein content, 15–25% polysaccharide, 5–6% total lipid, 6–13% nucleic acids, and 2.2–4.8% minerals (11). The Food and Drug Administration (FDA) has permitted GRAS certification (Generally Recognized as Safe) for Spirulina consumption and allowed to consume it as a food or food supplement (12). Consumption of Spirulina has potential health effects such as immunomodulation, antioxidant, anticancer, antiviral, and antibacterial activities and positive effects against malnutrition and anemia (13, 14). The addition of Spirulina in food has been seen as an emerging trend in several studies in the past decade (15–17).

Considering the higher prevalence of malnutrition in Tumkur district of Karnataka, Spirulina Foundation launched its program and distributed the spirulina chikki/granules to the children from 6 months to 6 years in Pavagada Taluk of Tumkur district. Before the launch of the program, baseline assessment was carried out, followed by intervention viz., distribution of spirulina chikkis/granules and endline assessment. It is hypothesized that nutritional supplementation in the form of spirulina chikki/granules could improve the nutritional status of malnourished children. With this background, the objective of the study is to evaluate the impact of Spirulina Chikki intervention on the nutritional status of children (6 months−6 years) in the Tumkur district, Karnataka, India.

## Materials and Methods

### Study Design and Participants

The study assessed nutritional status of the children (6 months−6 years) who consumed spirulina chikki/granules during the intervention period (September 2020–August 2021). Total 106 villages viz., 108 AWCs in nine circles was covered in Pavagada Taluk, Tumkur District. A cross-sectional study design was adopted to assess the changes in nutritional status among the children (after 12 months of intervention). The baseline and end-line assessment were carried out from September 2020 to August 2021, respectively. Children aged 6 months−6 years were the sample respondents. In the sample, all the Moderately Acute Malnourished (MAM) and Severely Acute Malnourished (SAM) children selected by the Spirulina Foundation for the intervention were covered. A pretested study tool was utilized to collect the information from the parents of the program beneficiaries. Informed written consent was obtained from the beneficiaries/subjects' parents/guardians for participation in the interview. Anthropometric measurements viz., Height, Weight, and Mid-Arm Circumference have been collected from 971 children and 838 children in baseline and endline assessment, respectively. The dropout rate of subjects was around 14 percent from baseline to end line. The information on children's health and nutrition status was collected from the mothers of beneficiaries/children.

### Sampling Design

A multi-stage sampling design was adopted for the study. Firstly, the Tumkur district was selected for the study. Secondly, Pavagada taluk was selected out of 10 taluks purposively due to the high prevalence of SAM/MAM children. Thirdly, all 106 AWCs from 106 villages (nine circles) from the taluk was chosen for the study. Fourthly, information from all the mothers/caretakers of program beneficiaries (6 months−6 years children) at the Anganwadi centers was collected for the study.

### Ingredients and Nutritional Values of Spirulina Chikki

Spirulina chikki consists of the following ingredients per KG: Ground Nut 500 g, Jaggery 245 g, Millet 200 g, Spirulina 50 g, and Cardamom 5 g. Approximate Nutritional value (per 100 g): Energy 425 Kcal, Protein 9.22 g, Fat 6.58, Carbohydrate 82.14 g, and Total sugar 49.5 g.

Approximate Nutritional value (per chikki.): Energy 42.5 Kcal, Protein 0.922 g, Fat 0.658, Carbohydrate 8.214 g, and Total sugar 4.95 g.

Recommended quantity of chikki bars/granules distributed to the various sample population is presented in Table 1. Chikki bars/granules was provided for a period of 1 year from September 2020–August 2021.

**Table 1:** Quantity of chikki bars/granules distributed to beneficiaries during intervention.

Age 2–6 years		
SAM children	4 Chikkis/day	1 Chikki = 10 g
MAM children	2 Chikkis/day	
06–23 months		
SAM children	2 Spoon granules/day	1 Spoon =5 g
MAM children	1 Spoon granules/day	

***Chikki** is ready to eat a traditional sweet snack (bars) that can be consumed by school children or pre-schoolers, or any other specific target group. **Granules** are small balls that can be consumed by children aged <2 years; basically, those who cannot chew the food items*.

### Anthropometric Measurements and Variable Description

The study has considered the four forms of malnutrition for children 6 months−6 years. We have considered four indicators Wasting (WHZ < −2SD), Stunting (HAZ < −2SD), and Underweight (WAZ < −2SD). Mid-Upper Arm Circumferences (MUAC) were categorized as per the WHO and UNICEF; normal (>12.5 cm), yellow (11.5–12.5 cm), and red (<11.5 cm) for malnutrition for children aged between 6 months and 6 years.

Each child was measured for height, weight and MUAC in the metric system, using the standardized technique recommended. Using a stadiometer (measuring rod), measured the height to an accuracy of 0.1 cm. Children were made to stand without footwear with the feet parallel and with heels, buttocks, shoulders and avoid touching the measuring rod, hand hanging by the sides. The head was at ease upright, with the top of the head making firm contact with the horizontal headpiece. The height of infants was measured using the infantometer. A convenient balance with a precision of 100 g was used to record the weight of the children. Children were directed to stand on the balance out with light clothing and without footwear and with feet apart and seeing straight. Mid-upper arm circumferences of children aged 6 months−6 years were measured using the MUAC tape endorsed by UNICEF.

A study has considered program-related variables along with anthropometric variables. The program-associated variables are heard about the spirulina foundation program, beneficiary of the program, frequency, and quantity of receiving Chikki/Granules, knowledge about the consumption patterns of Chikki/Granules and changes observed in terms of growth and feeding practice among the children.

### Statistical Analysis

Univariate and bivariate analysis was used to understand the frequency distribution of the variables. For the continuous variables, the mean and standard deviation was estimated. For the anthropometric measurements, WHO-recommended Anthro, AnthroPlus software and SPSS Version 25 Software was used.

## Results

Table 2 presents the awareness about the program among the beneficiaries. Most of respondents (97%) reported that they had heard about the program launched by the Spirulina foundation. Among them, 92% of the respondent said that they are the program beneficiaries.

**Table 2:** Awareness about the program among the beneficiaries.

	**Frequency (*N*)**	**Percent (%)**
**Heard about Spirulina foundation's program**		
Yes	810	96.7
No	28	3.3
**Total**	**838**	**100.0**
**Beneficiaries of the program**		
Yes	744	91.7
No	68	8.3
**Total**	**812**	**100.0**

Knowledge and consumption patterns of spirulina chikki/granules are described in Table 3. More than three-fourths of the children (78%) received Spirulina chikki, and 22% received granules. Almost all the beneficiaries (97%) had received spirulina chikkis/granules every month and the rest 3 percent every week. Furthermore, around two-thirds of the children received spirulina chikki/granules for 10 months and 24 percent for 9 months. In terms of the quantity received, 78% stated that they had collected four packets of spirulina chikki, and 19% said that they received one bottle of granule, and the rest 3.6% received two bottles of granules in the month.

**Table 3:** Spirulina chikkis/granules received and consumed by the beneficiaries.

**Indicators**	**Frequency (*N*)**	**Percent (%)**
**Child offered chikki/granules**		
Chikki bar	582	78.2
Granules	162	21.8
**Frequency of receiving chikkis/granules**		
Monthly	720	96.8
Weekly	24	3.2
**Duration of receiving chikkis/granules**		
Average months	9.4 ± 1.54	740
1–8 months	63	8.6
9 months	174	23.5
10 months	480	64.7
11–12 months	23	3.2
**Received chikki packets/granules bottles in 1 month**		
4 packets chikkis	578	77.8
One bottle granules	136	18.6
Two bottles granules	26	3.6
**Number of chikkis/granules spoon consumed by the child in a day**		
2 Chikkies/day	578	77.9
One spoon granule	127	17.3
Two spoon granules	35	4.8
**Chikki bars/granules consumed by the child**		
Average months	9.4 ± 1.54	739
1–8 months	61	8.3
9 months	168	22.6
10 months	490	66.3
11–12 months	21	2.8
**Proportion of chikkis/granules consumed by the child**		
All the chikki/granules	690	93.2
75% of the chikki/granules	27	3.6
50% of the chikki/granules	15	2.0
25% of the chikki/granules	06	0.8
None	02	0.3
**Chikkis shared with their siblings or other members**		
Yes	44	5.9
No	696	94.1

Regarding the mother's knowledge about the number of chikkis and amount of granules to feed the children, 78% of mothers informed two chikkis per day, 17% mothers reported one spoon granules, and around 5% mothers told two spoon granules in a day. And almost the same amount of chikkis and granules was consumed by the children. On average child consumes chikki/granules for 9.4 months. However, 66% of children consumed for 11–12 months. Majority of the children (93%) consumed all the chikki and granules. In contrast, 6% of the children shared chikkis/granules with their siblings.

Perception of the mothers on changes in the child's growth indicated that; a large majority (89%) of them reported that they had observed the change/growth in their child due to consumption of spirulina chikkis/granules. Approximately 50% of the mothers observed an increase in appetite, and 11% stated an increase in the child's weight. Mothers also reported that their children started eating increased quantity of food. It was evident that a majority (95%) of the children liked the taste of the chikki and asked for more chikkis/granules. A large proportion of the mothers reported (95%) their child eagerly ate the chikkis/granules (Table 4).

**Table 4:** Mothers perception about the changes in child's diet, and growth after consumption of chikkis/granules (*N* = 740).

**Variables**	**Frequency (*N*)**	**Percent (%)**
**Any change observed in child after consumption of chikki/granules**		
Yes	657	88.8
No	80	10.8
Don't know/can't say	03	0.4
**Changes observed in diet and growth**		
Increase in appetite	366	49.3
Increase in food intake	101	13.6
Increase in height	59	7.9
Increase in weight	131	17.7
No response	83	11.4
**Change in feeding pattern**		
Feeding more	601	81.3
Feeding less	11	1.5
No change	128	17.2
**Child liked the taste of the chikki/granules**		
Yes	703	95.1
No	32	4.3
Don't know/can't say	04	0.5
**Child demanded for more chikki/granules**		
Yes	673	90.9
No	65	8.8
Don't know/can't say	02	0.3
**Child ate the chikki/granules eagerly**		
Yes	700	94.6
No	36	4.9
Don't know/can't say	04	0.5

Anthropometric indicators show positive change over 1 year from baseline to end line assessment. The mean weight of the children improved from 9.76 to 11.1 Kg and height from 83.07 to 89.11 cm. Mid-Upper Arm Circumferences also exhibit an improvement from 13.19 to 13.8 cm in a 1-year intervention period. Further, results pointed out decline in Severely Wasted (WHZ < −3 SD) children from 9.5 to 13.1%, 3% decline in Wasted children (29.2–32.2%) at endline. In terms of Stunting, the result shows an improvement viz., with 4% (28.6–24.5%) decline in Severely Stunted (HAZ < −3SD) at endline. For the indicator Underweight, the result found a positive change in terms of reduction (6% at endline) in Severely Underweight children (WAZ < −3 SD) (30–23.9%) in the study (Table 5).

**Table 5:** Nutritional status of the children.

**Anthropometric indicator**	**Baseline**		**End line**	
	**Mean ±SD**	**Number**	**Mean ±SD**	**Number**
Weight	9.76 ± 2.2	930	11.1 ± 2.0	735
Height	83.07 ± 10.99	930	89.6 ± 11.0	735
MUAC	13.19 ± 0.94	930	13.8 ± 1.1	735
**Wasted**				
Severely wasted	9.5	88	13.1	96
Moderately wasted	29.2	272	32.2	236
Normal	61.3	570	54.8	402
**Stunted**				
Severely stunted	28.6	267	24.5	180
Moderately stunted	34.7	324	34.4	253
Normal	36.8	344	41.1	302
**Underweight**				
Severely underweight	30.0	281	23.9	152
Moderate underweight	46.2	433	50.2	319
Normal	23.9	224	25.9	165
**Total**	**100.0**	**930**	**100.0**	**735**

The differentials in prevalence of anthropometric measurement among gender presented in Table 6. Incline in Severely Wasted (WHZ < −3 SD) children was observed among male (3%; 13.5–16.5%) and female (4%; 5.9–10.2%) children at endline. However, incline in the prevalence of Wasted (WHZ < −2 SD) was higher among female children (7%) as compared to their male counterparts (1.5%). Further, the prevalence of Severely Stunting (HAZ < −3 SD) showed a positive change viz., decline was higher among male children's (8%) than their female counterparts (0.7%) at endline. A substantial proportion of the male children (9%) shifted from severely underweight to moderately underweight; however, this shift was lower (3%) among the female children at endline.

**Table 6:** Nutritional status of the children.

**Anthropometric measurements**	**Baseline**			**End line**		
	**Boy (%)**	**Girl (%)**	**Overall**	**Boy (%)**	**Girl (%)**	**Overall**
**Wasted**				
Severely wasted	13.5	5.9	9.5	16.5	10.2	13.1
Wasted	32.0	26.8	29.2	30.6	33.5	32.2
Normal	54.6	67.2	61.3	52.9	56.3	54.8
**Stunted**				
Severely stunted	34.8	23.0	28.6	27.1	22.3	24.5
Stunted	32.0	37.0	34.7	32.1	36.5	34.4
Normal	33.2	40.0	36.8	40.9	41.3	41.1
**Underweight**				
Severely underweight	34.5	25.9	30.0	25.4	22.5	23.9
Moderately underweight	46.8	45.6	46.2	49.2	51.1	50.2
Normal	18.7	28.5	23.9	25.4	26.4	25.9

The Mid-Upper Arm Circumferences of the children showed constructive improvements. At endline, the overall proportion of Severe Acute Malnutrition (SAM) declined from 5.3 to 0.6% and Moderately Acute Malnutrition (MAM) from 23 to 9%. Due to these improvements, the proportion of normal children incline from baseline (72%) to end line (91%). These improvements were observed among both male and female children; percentage of male SAM children declined to 0.8 from 3.8% and female MAM children to 0.4 from 6.6% at endline from baseline, respectively (Table 7).

**Table 7:** Mid-arm circumferences of children aged 6 months−6 years.

**MUAC**	**Male (%)**	**Female (%)**	**Overall**	**Color coding as based on measuring tape**
**Baseline**				
SAM	3.8	6.6	5.3 (51)	Red (<11.5 cm)
MAM	24.9	21.2	23.0 (223)	Yellow (11.5–12.5 cm)
Normal	71.3	72.2	71.8 (697)	Normal (>12.5 cm)
**End line**				
SAM	0.8	0.4	0.6 (0.6)	Red (<11.5 cm)
MAM	8.4	9.4	8.9 (8.9)	Yellow (11.5–12.5 cm)
Normal	90.9	90.1	90.5 (758)	Normal (>12.5 cm)

The nutritional status viz., Stunting, Wasting, and Underweight across duration of the consumption of chikkis and granules is presented in Table 8. The children who consumed chikkis/granules for at least 9 months regularly had the lowest prevalence (11.7%) of Severely Wasted (WHZ < −3 SD), and the prevalence was the highest (18.3%) among those children who consumed for 1–8 months. The proportion of Severely Stunted children (21.9%) and Severely Underweight children (21.7%) were the lowest among those children who consumed chikkis/granules for 10 months.

**Table 8:** Nutritional status across duration of the consumption of chikkis and granules.

**Anthropometric measurements**	**1–8 months**	**Up to 9 months**	**Up to 10 months**	**11–12 months**	**Overall**
**Wasted**					
Severely wasted	18.3	11.7	13.0	14.3	13.2
Wasted	26.7	37.4	31.1	23.8	32.0
Normal	55.0	50.9	55.9	61.9	54.9
**Stunted**					
Severely stunted	32.8	27.6	21.9	33.3	24.4
Stunted	31.1	34.4	35.5	28.6	34.7
Normal	36.1	38.0	42.7	38.1	41.0
**Underweight**					
Severely underweight	27.1	28.4	21.7	31.6	23.9
Moderately underweight	50.0	50.4	50.7	47.4	50.5
Normal	22.9	21.3	27.6	21.1	25.6

## Discussion

The high prevalence of child malnutrition in India has been considered a significant challenge as the proportion of stunted and underweight children are considerably higher in India. To address the issue of malnutrition among children, the Spirulina Foundation launched the program under which they had trained and empowered local self-help group (SHG) women to cultivate spirulina and make Spirulina Fortified Chikkis (SFCs)/granules. The program's overall aim is to improve the nutrition level/status of young children's overall health by providing the Spirulina Chikkis consistently in the study area. Institute of Health Management Research (IIHMR), Bangalore, is a nodal agency/technical partner for conducting the impact assessment to identify the changes in nutrition status of the children. The present study measured the impact of Spirulina Chikki intervention on the nutritional status of the children (6 months−6 years) in Pavagada Taluk, Tumkur district of Karnataka, India.

The result reveals that a majority of the mothers reported that they had observed the change/growth in their child after consumption of spirulina chikkis/granules. Approximately 50% of the mothers observed an increase in appetite, and eleven percent stated an increase in the child's weight. A study conducted in Burkina Faso reveals that the treatment with Spiruline plus Misola have synergically favor the nutrition recovery better than the simple addition of protein and energy intake (18). The spirulina is beneficial and essential for the growth of infants and suitable for children, especially in the growth phase. It helps in case of general weakness and anemia. Due to its nutritional value, WHO considered spirulina as a superfood for the future (14**)**. The mean weight of the children improved from 9.76 to 11.1 Kg and height from 83.07 to 89.11 cm. Another critical indicator mid-upper arm circumferences also exhibit an improvement from 13.19 to 13.8 cm at endline. Evidence from the study conducted in Bellary district of Karnataka observed that 47 and 68% reduction in malnutrition among children who received 1 and 2 g of Spirulina, respectively, as compared to little change in two control groups (*p* < 0.05). In 2 g Spirulina arm, an increase in mean weight of 1.25 g/kg/day (*p* < 0.01); and maximum weight gain of 7.3 g/kg/day (19).

The present study showed a slight upsurge in Severely Wasted (WHZ < −3 SD) and Wasted (WHZ < −2 SD) children. However, results of Stunting showed the improvement viz., decline of 4% (from 28.6% baseline to 24.5% endline) in Severely Stunting (HAZ < −3 SD) at endline. And study observed a positive change viz., 6% reduction in Severely Underweight children (WAZ < −3 SD) (from 30% baseline to 23.9% endline) at endline. A study also documented that the Spirulina is found to be the best alternative dietary supplement to the malnutrition. Spirulina is a safe food with absolutely no side effects, and it is a comprehensive bundle of macro and micronutrient (20–23).

In 1992 WHO has declared Spirulina as “Best food for future” to redress malnutrition especially in children (24). A study based on 50 samples confirmed that the weight-for-age *Z*-scores and weight-for-height *Z*-scores increased significantly in the intervention group (16). Weight for age *z*-score data a significant 44% reduction in malnutrition in mission data was validated by study conducted in Bellary district of Karnataka (19). Upsurge in Severely Wasted (WHZ < −3 SD) children observed more among male than female children over the intervention period. An incline in the prevalence of Wasted children were higher among the girl children than male children. The prevalence of Severely Stunted showed positive change, eight percent among male and around one percent among female children. A substantial proportion of the male children shifted from Severely Underweight to Moderately Underweight category. A Zambian study that examined the efficacy of spirulina on malnourished children found that 10 g of spirulina daily intake leads to improvement by producing 0.29 elevated points in the height-for-age *z*-score (HAZ). Also, the weight-for-age z-score (WAZ) and the Mid-Upper Arm Circumference *z*-score (MUACZ) did not show a significant difference. However, treated children showed a more extensive improvement by 0.09 and 0.38 points, respectively (17).

A substantial decrease in proportion of Severe Acute Malnutrition (SAM) and Moderate Acute Malnutrition (MAM) children was observed at endline. Due to these improvements, the proportion of normal children inclined from 72 to 91%. The improvements were observed among both male and female children. The children who consumed chikkis/granules for at least 9 months regularly had the lowest prevalence (11.7%) of Severely Wasted (WHZ < −3 SD), and the prevalence was the highest (18.3%) among those children who consumed for 1–8 months. The proportion of Severely Stunted children (21.9%) and Severely Underweight children (21.7%) were the lowest among those children who consumed chikkis/granules for 10 months. A study by Ramesh et al. (25) too revelated a significant increase in anthropometric measurements and Hemoglobin, serum ferrtin, serum zinc, serum protein and serum albumin levels in the study sample after 6 months of intervention (25).

## Conclusions

The supplementation with spirulina into a snack bar (known as chikki) and granules was well-accepted by the caretakers/mothers in the study sites. Consumption of spirulina chikki/granules for more than 9 months had impacted the nutritional status of the children positively. Strengthening the health literacy among the beneficiaries' families about the importance of spirulina chikki is critical for acceptance and successful of the program. The approach of utilizing algae as a source of alternative food supplements in the form of spirulina into a snack bar (known as chikki) and granules can represent one of the most promising approaches in the long term to address malnutrition in the developing country like India. The intervention shall be considered for incorporation into the government existing nutritional program for its sustainability along with local ownership/local production units and microeconomics. The world challenged by severe economic crises, which make the resources for development more uncertain, such endeavors seem more crucial than ever.

### Limitations of the Study

Only one Taluk (area) of Tumkur district was covered in this study. Therefore, we cannot generalize the findings for the entire district. Also, the present study had included the children (6 months−6 years) who were enrolled in the Anganwadi centers and program beneficiaries of the Spirulina Foundation program.

## Data Availability Statement

The raw data supporting the conclusions of this article will be made available by the authors, without undue reservation.

## Ethics Statement

The studies involving human participants were reviewed and approved by Women and Child Development, Tumkur. Written informed consent to participate in this study was provided by the participants' legal guardian/next of kin.

## Author Contributions

GK and RS developed the questionnaire. GK collected the data, contributed to acquisition of data, statistical analysis of data, and wrote the manuscript. RS and UM enhanced the questionnaire, contributed to the concept and design of the article, literature searches, and critically revised the manuscript. All authors read and approved the final manuscript.

## Funding

This study was supported by Bhoruka Charitable Trust.

## Conflict of Interest

The authors declare that the research was conducted in the absence of any commercial or financial relationships that could be construed as a potential conflict of interest.

## Publisher's Note

All claims expressed in this article are solely those of the authors and do not necessarily represent those of their affiliated organizations, or those of the publisher, the editors and the reviewers. Any product that may be evaluated in this article, or claim that may be made by its manufacturer, is not guaranteed or endorsed by the publisher.
